# Long-term brain changes in bipolar disorder

**DOI:** 10.1192/j.eurpsy.2022.416

**Published:** 2022-09-01

**Authors:** M. Landén, B. Liberg, C. Abé

**Affiliations:** 1 Gothenburg University, Institute Of Neuroscience And Physiology, Gothenburg, Sweden; 2 Karolinska Institutet, Dept Of Clinical Neuroscience, Stockholm, Sweden

**Keywords:** neuroprogression, longitudinal, Neuroimaging, bipolar disorder

## Abstract

**Introduction:**

The term “neuroprogression” imply that bipolar disorder (BD) progressively worsens for some patients and accompanying neuroanatomical changes. BD has indeed been associated with cortical and subcortical brain abnormalities. But cross-sectional studies cannot determine whether the observed brain alterations reflect static premorbid traits or whether they result from progressive changes during the course of illness.

**Objectives:**

The aims of this series of studies were to determine if progressive brain changes occur in bipolar disorder, and if so, what the drivers of these changes are.

**Methods:**

We addressed these questions in the St. Göran cohort – a longitudinal study where patients and controls undergo structural magnetic resonance imaging (MRI) scans at baseline and after 7 years. We have also conducted a longitudinal multicenter study within the ENIGMA consortium including 307 patients and 925 healthy controls scanned at two time points.

**Results:**

We addressed these questions in the St. Göran cohort – a longitudinal study where patients and controls undergo structural magnetic resonance imaging (MRI) scans at baseline and after 7 years. We have also conducted a longitudinal multicenter study within the ENIGMA consortium including 307 patients and 925 healthy controls scanned at two time points.

**Conclusions:**

BD is associated with some (accelerated ventricular enlargement) but not global progressive brain changes (change in cortical structures do not differ from controls). Occurrence of manic episodes is, however, associated with accelerated cortical thinning over time. These results highlight the importance of preventing the potentially toxic effects of manic episodes and might explain why some patients experience worsening cognitive function.

**Disclosure:**

ML has received lecture honoraria (unrelated to this topic) from Lundbeck pharmaceuticals.

